# Anion-Dependent Redox Pathways Governing Water Splitting in Superconcentrated Lithium Electrolytes

**DOI:** 10.1021/acsphyschemau.6c00037

**Published:** 2026-04-22

**Authors:** Sagar Ingavale, Pawin Iamprasertkun

**Affiliations:** † School of Bio-Chemical Engineering and Technology, Sirindhorn International Institute of Technology, 37698 Thammasat University, Klong Luang, Pathum Thani 12120, Thailand; ‡ Research Unit in Sustainable Electrochemical Intelligent, 37698 Thammasat University, Klong Luang, Pathum Thani 12120, Thailand

**Keywords:** “water-in-salt”, Oxygen evolution reaction, Hydrogen evolution reaction, Platinum, Voltage window

## Abstract

Electrochemistry involving superconcentrated electrolytes has rapidly gained attention as a transformative approach in electrochemical processes. Superconcentrated electrolytes have emerged as a groundbreaking solution in electrochemical applications, recognized for their nonflammable nature, ecofriendly composition, and expanded electrochemical stability window compared to conventional dilute aqueous electrolytes. The electrochemical hydrogen evolution reaction (HER) and oxygen evolution reaction (OER) are fundamental processes in energy conversion and storage technologies. This study investigates the electrochemical activity of platinum electrodes in lithium-based electrolytes for understanding the electrochemical characteristics from low to high concentration. The HER and OER kinetics in superconcentrated electrolytes were performed to address challenges related to stability and efficiency in high-concentration ionic environments. Highly concentrated electrolytes characterized by their unique solvation structure and extended electrochemical stability potential window offer promising avenues for enhancing energy storage and conversion applications. Through cyclic voltammetry and linear sweep voltammetry, we analyze key parameters such as redox reactions, overpotentials, and kinetic reactions. This study explores the interaction between platinum and highly concentrated aqueous electrolytes to elucidate its influence on catalytic performance. The results provide insights into optimizing platinum-based electrocatalysis for next-generation sustainable energy solutions, highlighting the role of electrolyte composition in dictating reaction kinetics, potential window, and overall electrochemical performance.

## Graphical abstract

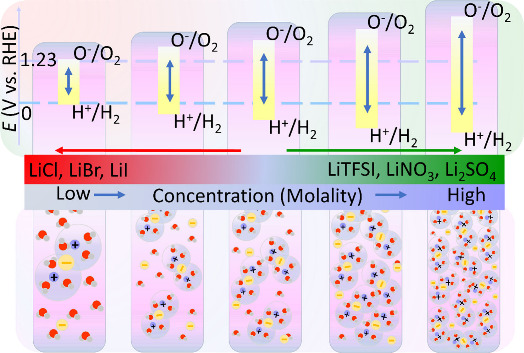

## Introduction

Superconcentrated electrolytes, encompassing both nonaqueous and aqueous electrolytes, have sparked significant interest due to their potential application in electrochemical technologies, including energy storage and conversion systems.^1^ A superconcentrated electrolyte is an innovative approach to electrochemical energy storage and conversion, as it is based on an aqueous system which is safer compared to nonaqueous electrolytes.^2^ In energy conversion, MOH (M = Li, Na, K) - added “water-in-salt” electrolytes, especially with Li^+^ cations, enhance OER performance on Fe–N–C catalysts by promoting OH^–^ availability and enabling dual Tafel pathways through irreversible oxidation of adsorbed species.^3^ Aqueous electrolytes offer superior safety due to water’s nonflammable nature, thermal stability, and low toxicity compared to hazardous organic solvents.^4,5^ In conventional aqueous electrolytes, the electrochemical stability window (1.23 V) makes a narrow potential window. The electrochemical stability of an electrolyte solution, known as its “potential window”, depends on the solvent’s oxidation and reduction limits. If dissolved solutes remain stable within this range, they avoid decomposition. Water, the most common electrochemical solvent, has a narrow thermodynamic potential window of 1.23 V. The electrochemical stability window is limited by the thermodynamic onset of water splitting into H_2_ and O_2_. This restricts high-voltage applications due to unwanted gas evolution beyond that range.^6^

Superconcentrated electrolytes have gained attention for their ability to dissolve large amounts of salt in minimal water, forming highly concentrated solutions. Kang Xu et al.^7^ introduced a notable example of 21 mol kg^–1^ (21 m) aqueous lithium bis­(trifluoromethane) sulfonamide (LiTFSI), which features a salt-to-water molar ratio (Li^+^/H_2_O) of 1:2.6. This composition enhances electrochemical stability, offering a potential window of around 3 V, which significantly boosts the aqueous battery energy density. Bélanger et al.^8^ investigated the capacitance of MnO_2_-based composite electrodes in dilute and highly concentrated electrolytes and found enhanced capacitance and a broader stability window in highly concentrated 5 M LiTFSI. Water in salt electrolytes (WiSEs) offer a safer and more cost-effective alternative to organic electrolytes.^9^ Researchers have explored various salts including LiTFSI, ZnCl_2_, NaClO_4_, NaNO_3_, and KCF_3_SO_3_ for WiSE systems, demonstrating their ability to expand the voltage window and improve energy storage applications.^2,10^ These advancements continue to expand the potential applications of highly concentrated electrolytes in energy storage. Beyond energy storage applications, WiSEs are developing as advanced electrolytes for nitrogen and CO_2_ reduction reaction.^11,12^

The electrochemical water splitting reaction encompasses the HER and the OER. Theoretically, to drive the entire reaction, it requires a potential difference of 1.23 V between the anode and cathode.^13^ Due to the sluggish kinetics of both HER and OER, substantial overpotentials are required to produce significant current density, resulting in relatively low energy conversion efficiency. Several electrochemical applications, including aqueous rechargeable lithium and sodium batteries and lead-acid rechargeable batteries, got affected with respect to potential window.^14,15^ To tackle this issue, several researchers attempted to use concentrated aqueous electrolytes to improve the potential window.^16,17^ These findings suggest that the electrolyte concentration influences the potential window of water. However, the underlying mechanism remains inadequately understood as increasing concentration is not in constant growth. Despite substantial efforts to develop WiSEs for storage applications, their stability and instability remain poorly understood. This limitation arises from the scarcity of research on the distinct contributions of HER and OER. Yang et al. quantitatively evaluated the influence of thermodynamics, kinetics, and interface layers on the apparent HER activity in 20 m LiTFSI electrolyte.^6^ Anku and Tharangattu analyzed the high-concentration Li^+^ electrolytes to enhance the electrocatalytic HER performance of carbon nanotubes (CNTs), with both MWCNTs and SWCNTs exhibiting similar trends—contrary to the behavior observed with platinum.^18^ Another work demonstrated achievement of low-overpotential OER using an Fe–N–C model surface, conducted in a highly concentrated metal trifluoromethanesulfonate environment.^3^ Marion et al.^19^ proposed the passivation layer, formed through electrode oxidation, salt decomposition, and precipitation, consists of fluorinated products like LiF and CF_3_, effectively preventing further water or carbon oxidation and leading to a higher potential shift. Most of the studies have primarily focused on LiTFSI electrolytes; however, “water-in-LiTFSI” has been costly and exhibited low ionic transport.^20^ Thus, other lithium-based electrolytes need to be investigated for energy storage and conversion applications.

This study explores the potential window and redox behavior of a platinum (Pt) electrode in concentrated lithium-based electrolyte solutions. The reliable electron transfer, chemical robustness, and inherent catalytic power of the platinum electrode make it indispensable across diverse electrochemical HER/OER and fuel cell applications. Here, the electrolytes were formulated using various lithium-based salts, including lithium bis­(trifluoromethanesulfonyl)­imide (LiTFSI), lithium nitrate (LiNO_3_), lithium chloride (LiCl), lithium bromide (LiBr), lithium iodide (LiI), and lithium sulfate (Li_2_SO_4_). The choice of electrolyte plays a pivotal role in enabling cost-effective device development, as current findings indicate that certain electrolytes exhibit corrosive behavior, some function effectively as redox additives, while others are well-suited for ″water-in-salt″ applications. HER and OER are the basic reactions that occur during reaction in electrochemical processes, especially in an aqueous electrolyte. Thus, by analyzing HER/OER kinetics in high-concentration ionic environments, the research addresses stability and efficiency challenges. Key parameters for these redox reactions and reaction overpotentials are evaluated to assess the impact of platinum in these concentrated aqueous systems using cyclic voltammetry (CV) and linear sweep voltammetry (LSV) techniques.

## Results and Discussion

Lithium-based electrolytes are studied extensively, especially in high-concentration aqueous systems, for several compelling reasons including high ionic conductivity, wide electrochemical stability window, and compatibility with platinum and other noble metals. Electrolyte solutions were prepared based on molality (mol kg^–1^), abbreviated as [m] (Table S1). As a result, the physical properties of the prepared electrolyte, including pH and electric conductivity, were initially analyzed. pH plays a significant role in governing the reaction pathways and kinetics of electrochemical processes that involve proton–electron coupling.^21^ Accordingly, understanding how pH influences reaction activity and selectivity is essential for enhancing overall reaction efficiency.^21^ The pH of the electrolyte is highly sensitive to concentration, as shown in Figure S1a, which directly influences the corrosion behavior of the cell when interacting with a metal substrate. pH reflects the activity, rather than simply the concentration, of hydrogen ions. As the ionic strength increases through the addition of salts, ion interactions cause activity coefficients to decrease, which raises the measured pH despite constant hydrogen ion concentration. When concentrations are high or multiple ionic species are present, these interactions become nonlinear and make pH behavior more complex to predict.^22^ High pH and low pH can easily corrode the electrode due to the ability to dissolve the protective oxide of the metal and provide the proton to react with the metal, respectively; these lead to corrosion.^23^ Consequently, the high-concentration electrolyte with pH close to neutral will be more preferable to minimize the corrosion process. Among all electrolytes, LiTFSI and LiNO_3_ electrolytes were able to maintain a near-neutral pH when concentration increased, making it a preferable choice for minimizing corrosion. Also, Li_2_SO_4_ maintains an almost neutral pH due to the minimal difference in the concentration. In contrast, other salts, such as LiBr and LiI, transitioned from a basic to a neutral state upon reaching their maximum concentration. The LiCl remained nearly neutral at a low concentration, but above 10 m, it exhibited acidity under ″water-in-salt″ conditions. Consequently, it is recommended to use anticorrosion substrates including carbon allotropes, metal sulfides/nitrite/phosphite, etc. while using halide- and nitrate-based electrolytes to avoid potential degradation.

The ionic conductivity of the electrolyte depends on the concentration of dissolved ions. In dilute electrolytes, more ions become available to carry an electric charge. In this region, conductivity increases almost proportionally with concentration because ions freely move without much interference. Further, in a moderately concentrated electrolyte, the conductivity continues to rise, but now ion interactions begin to play a role. More ions mean a higher possibility of ion–ion interactions, which can slightly hinder their movement. In concentrated electrolyte solutions, ion pairing reduces the number of free charge carriers, which lowers the overall conductivity. Thus, conductivity generally increases with concentration due to the higher number of dissolved ions, but at very high concentrations, ion interactions can lead to decreased mobility, slightly reducing conductivity.^24,25^ A similar trend is observed in the prepared electrolytes (Figure S1b). In most of the electrolytes, the higher electrical conductivity is observed at the 5 and 10 m concentrations compared to 1 and 20 m electrolytes. The lithium halides, 5 m LiBr, 5 m LiBr, and 3 m LiI, demonstrated higher conductivity compared to other lithium-ion counterparts. At superconcentrated conditions, LiNO_3_ displays better conductivity compared to LiCl and LiTFSI. In 20 m LiNO_3_, the highest ionic conductivity (93 ± 0.05 mS cm^–1^) was observed, which is almost 12 times higher than 20 m LiTFSI. Notably, along with higher conductivity, LiNO_3_ electrolyte maintains pH almost to neutral. In addition, as the salt concentration increases, the mole ratio of water to Li^+^ ions decreases markedly due to the reduced amount of free water, which becomes increasingly coordinated with the ions. Consequently, a “water-in-salt” electrolyte is expected to form at high concentrations. In our prior study, we found that electrolyte viscosity tends to increase with higher concentration.^20^ Recent studies have correlated electrolyte diffusion with concentration by emphasizing the role of viscosity. Paul Robin^26^ demonstrated that electrostatic correlations induce viscous dissipation, reducing diffusion at higher concentrations. Complementarily, Prerna & Kant^27^ developed a soft ionic atmosphere model that quantitatively links molar conductivity, diffusion coefficients, and viscosity, confirming that entropy-driven ionic atmosphere expansion suppresses diffusion as concentration rises.

Fourier transform infrared spectroscopy-attenuated total reflectance (ATR-FTIR) spectroscopy was carried out from 400 cm^–1^ to 4000 cm^–1^ in electrolytes with increasing concentration (Figure 1 and Figure S2). The FTIR spectra of all electrolytes reveal key insights into the molecular interactions between water and salts at different concentrations. The spectra display characteristic peaks corresponding to H–OH bending (∼1600 cm^–1^) and stretching (∼3400 cm^–1^) vibrations. At the beginning, the FTIR spectra of water revealed H–OH bending of ∼ 1636 cm^–1^ and stretching of ∼ 3281 cm^–1^. At low concentration in all electrolytes (1 m), the H–OH bending and stretching remains the same, as the water structure remains unchanged. As concentration increases to 5 m or above, the shift in absorbance peaks was observed in all higher concentrations, as Li^+^ ions start to begin to disrupt the hydrogen bonding network, leading to significant shifts. At maximum concentration, a strong ion–water interaction significantly alters the vibrational modes, leading to peak shrinking and intensity variations.

**Figure 1:**
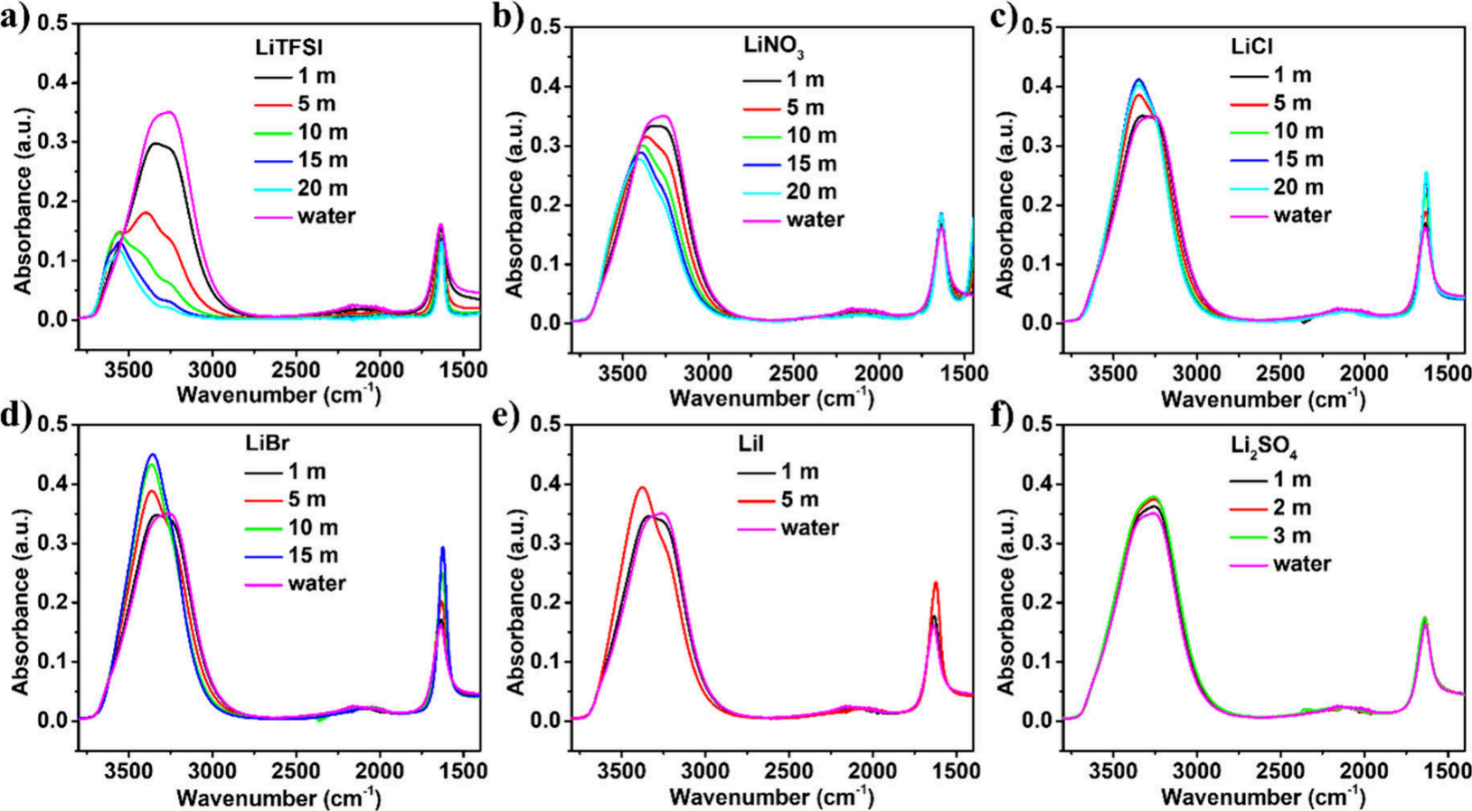
ATR-FTIR spectra of H–OH bending and H–OH stretching, of different concentrations of a) LiTFSI, b) LiNO_3_, c) LiCl, d) LiBr, e) LiI, and f) Li_2_SO_4_ electrolyte.

In every electrolyte, the red shift is observed with respect to water except in the case of Li_2_SO_4_, where a blue shift was seen (a stronger interaction between sulfate ions and water, slightly shifting the bending mode). Ion-dipole interactions, hydrogen bond disruption, and reduced free water molecules are the main reasons for shift in FTIR peaks in all prepared electrolytes. Li^+^ ions strongly interact with water molecules, altering their vibrational modes and reducing the intensity of characteristic peaks. In the case of LiTFSI, the H–OH bending at 1 m electrolyte was observed similar to water (1636 cm^–1^); afterward, it red-shifted with increasing concentration, and it was found ∼1633 cm^–1^ in 20 m electrolyte. The red shift in LiTFSI suggests a subtle weakening of the hydrogen-bonding network due to Li^+^ interactions. Li^+^ forms solvation shells, modifying the electron density around water molecules, leading to a small decrease in the bending frequency. In LiTFSI solutions, many water molecules are bound to Li^+^, reducing their contribution to absorbance. Contrasting to LiTFSI, the halides (LiCl, LiBr, and LiI) can promote specific hydrogen-bonding structures, leading to stronger absorption features.

In water, the 3281 cm^–1^ peak is associated with the O–H stretching, which is typically broad due to hydrogen bonding. The shift in the H–OH stretching frequency from 3281 cm^–1^ in pure water to 3554 cm^–1^ in a 20 m LiTFSI solution is primarily due to changes in hydrogen bonding interactions and the solvent structure. In highly concentrated LiTFSI solutions, Li^+^ ions interact strongly with water molecules, disrupting the hydrogen bond network. This leads to a blue shift in the OH-H stretching frequency. The presence of TFSI anions alters the local environment of water molecules, weakening hydrogen bonding and increasing the vibrational frequency.^28^ Besides, at high salt concentrations, water molecules form small clusters rather than an extended hydrogen-bonded network, further contributing to the frequency shift due to strong hydration effects and unique sulfate interactions. Unlike LiTFSI or LiNO_3_, Li_2_SO_4_ has highly charged sulfate ions that strongly interact with water molecules, reinforcing hydrogen bonding and lowering the O–H stretching frequency. Sulfate ions tend to form stable hydration shells, reducing the availability of free water molecules and shifting the peak downward. The divalent sulfate anion (SO_4_^2–^) has a stronger electrostatic field than monovalent anions like Cl^–^ or Br^–^, further stabilizing water’s hydrogen bonding network. The decrease in the FTIR peak intensity corresponds to Li^+^ bound to water molecules, which reduces their contribution to absorbance. The FTIR absorbance peak shift in 3 m Li_2_SO_4_ to 3268 cm^–1^ is lower than other lithium salts.

The wettability of the electrode–electrolyte interphase ensures sufficient contact of the electrode surface with the electrolyte ion from water-in-salt electrolytes, thereby optimizing water-splitting performance. Electrolyte wettability was evaluated by measuring the contact angle (CA) of the electrolyte on the surface of the platinum electrode. A droplet of each electrolyte was deposited onto the electrode, and the CA (θ) was defined as the angle between the horizontal line of the liquid–solid interface and the tangent line of the gas–liquid interface. Based on θ, the surfaces were classified as superwetting (θ < 10°), wetting (10° < θ < 90°), and nonwetting (θ > 90°). As shown in Figure S3, LiTFSI and LiCl showed a significant decrease in contact angle with increasing concentration (from 70.03° to 46.31° and 83.45° to 67.03°, respectively), reflecting enhanced wettability. LiNO_3_ and Li_2_SO_4_ exhibited only minor decreases (90.14° to 86.56° and 87.46° to 85.98°), suggesting limited changes in spreading behavior. LiBr showed virtually no variation (85.69–85.87°), while LiI displayed an increase (83.11–87.17°), indicating reduced wettability at higher concentration. Overall, these results demonstrate that different electrolytes exhibit distinct wettability trends on platinum, with some enhancing surface spreading as the concentration increases, while others show negligible or even opposite effects.

The electrochemical measurements were conducted by using a well-calibrated three-electrode setup with nitrogen-saturated electrolytes. All current densities were normalized to the active surface area of platinum, and potentials were referenced to RHE. The electrochemical measurements detail is given in Supporting Information. CVs and LSVs were employed to test the OER and HER catalytic activities of the platinum electrode in various electrolytes at room temperature. Figure 2 reveals CVs in prepared lithium-based salts with increasing concentrations ranging from 1 to 20 m at a scan rate of 100 mV s^–1^. The CV was recorded by scanning the potential in positive and negative directions to achieve an arbitrarily high current density of about 1–10 mA/cm^2^. The voltammogram for a Pt electrode displays an onset of oxidation presumably corresponding to oxygen evolution at about 2.14 V, which is shifted approximately to 2.39 V when the concentration of LiTFSI salt is increased from 1 to 20 m. The CV potential windows of LiTFSI, LiNO_3_, and Li_2_SO_4_ were expanded with increasing concentration. However, the CVs of LiCl, LiBr, and LiI become compressed due to their redox-active properties.

**Figure 2:**
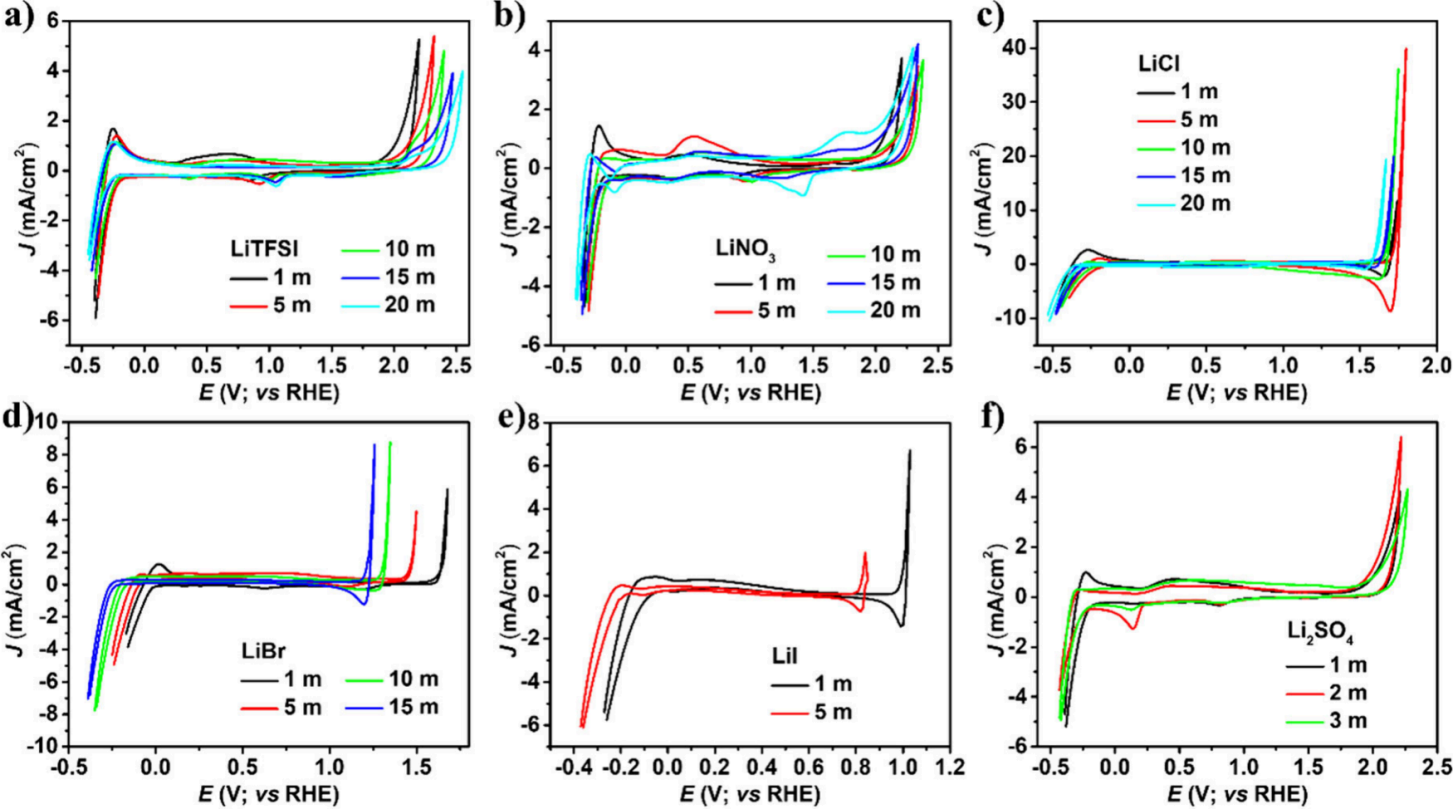
Cyclic voltammograms of Pt electrode at scan rate 100 mV s^–1^ in different concentrations of a) LiTFSI, b) LiNO_3_, c) LiCl, d) LiBr, e) LiI, and f) Li_2_SO_4_ electrolytes.

LiI exhibits an average redox voltage potential window close to 1.2 V, which is the lowest among prepared lithium-based electrolytes. The iodide oxidation is observed from 1.0 V (1 m) to 0.835 V vs RHE (5 m), with increasing concentration. The LiI concentration appears to influence the redox process, causing a noticeable delay in the reaction kinetics. In case of LiBr, similar bromide oxidation and reduction were seen with redox potential windows close to 2.0 V vs RHE. Increasing the concentration of redox-active salts such as LiBr and LiI enhances the redox reaction, allowing oxidation and reduction processes to proceed more rapidly compared to lower concentrations. The voltage window is slightly compressed, indicating that the concentration primarily influences reaction kinetics rather than alters the electrochemical potential range. In the case of LiCl, the voltage window changed by approximately 0.15 V. However, this electrolyte is highly corrosive, making corrosion prevention essential. To mitigate its effects, either an alternative substrate or corrosion protection techniques must be implemented to safeguard the electrode material.

In the present work, we prioritized analyzing the redox behavior of the Pt electrode and factors responsible for the potential window. The potential windows are influenced by water electrolysis reactions, specifically the OER and HER (affecting the storage potential window). The onset potential for OER/HER was defined at a current density of 1 mA/cm^2^ in LSV measurements conducted at a scan rate of 25 mV s^–1^ (Figure 3 (a)). HER reveals a steady trend across lithium-based electrolytes due to its occurrence at negative potentials, where water activity dominates and anion adsorption is minimal. In contrast, the OER occurs at positive potentials, making it highly sensitive to anion adsorption. Water activity plays a lesser role, and surface interactions can significantly impact the reaction kinetics. Thus, the OER onset potentials vary with different lithium salts, unlike HER. The overall potential window is determined from the OER/HER and the redox onset potential from LSVs. Figure 3 (b) illustrates how these potential windows vary depending on water and salt concentrations, revealing two prominent trends. First, the electrochemical windows in LiTFSI, LiNO_3_, and Li_2_SO_4_ electrolyte solutions are noticeably broader than those in LiCl, LiBr, and LiI electrolyte solutions at various concentration levels. The LiTFSI and LiNO_3_ demonstrated an almost similar potential window with increasing concentration. Also, it shows a similar window at low concentration as Li_2_SO_4_. It proves that this type of salt does not significantly influence the electrochemical window. Instead, the window size exhibits a linear dependence on water concentration-decreasing water content (i.e., increasing salt concentration) leads to an expanded electrochemical window. As opposed to this, redox-active ions in LiCl, LiBr, and LiI impacted the potential window. Despite decreasing water content, the redox reactions are favorable, which suppressed the potential window.

**Figure 3:**
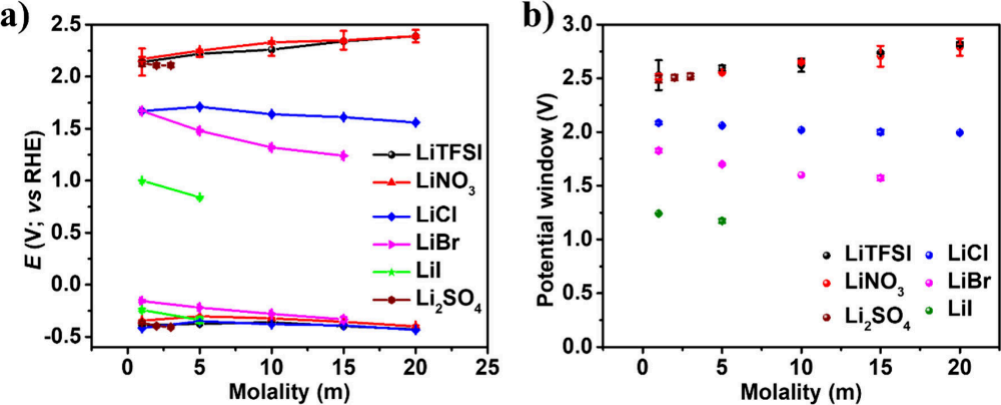
(a) Onset potential of the platinum electrode in lithium-based electrolytes at the current density 1 mA/cm^2^ from LSVs. (b) Potential window width dependence on water concentration (Potentials were selected from the onset potential at the current density 1 mA/cm^2^ from LSVs).

Instead of relying on subjective current density criteria to define the working potential range of the electrolytes, we adopted a more systematic approach. Specifically, we utilized electrochemical impedance spectroscopy (EIS) to identify the potential window within which the system exhibits a purely capacitive behavior, thereby defining the true electrochemical stability range of the electrolytes (Figures S4 & S5). The capacitance of the platinum electrode is determined by identifying the potential at which the phase angle in the Bode plot approaches −90°, indicating a predominantly capacitive response.

In an ideal capacitor, the phase angle is approximately −90°, indicating purely capacitive behavior. In redox-active systems, this angle shifts toward 0°, typically ranging between −45° and −70°, due to the influence of faradaic reactions. These reactions introduce resistive components, making the system a combination of double-layer capacitance and charge transfer resistance.^29^ In the present study, the phase angle observed between −90° and 0° for LiTFSI, LiNO_3_, LiCl, LiBr, and Li_2_SO_4_ on a platinum working electrode indicates typical capacitive behavior dominated by double-layer formation (Figures S6 & S7).

The concentrations of 1 m and the maximum concentration of each electrolyte were used to investigate their impact on the electrochemical behavior of a platinum electrode. As shown in Figure 4, increasing the concentrations of LiTFSI, LiNO_3_, and Li_2_SO_4_ results in a broader potential window derived from the purely capacitive response of the system. In contrast, a narrowing of the potential window is observed with LiCl, LiBr, and LiI electrolytes (Figure 4). This behavior corresponds to the redox activity of the halide ions and is consistent with the results obtained from linear sweep voltammetry (LSV) analyses.

**Figure 4:**
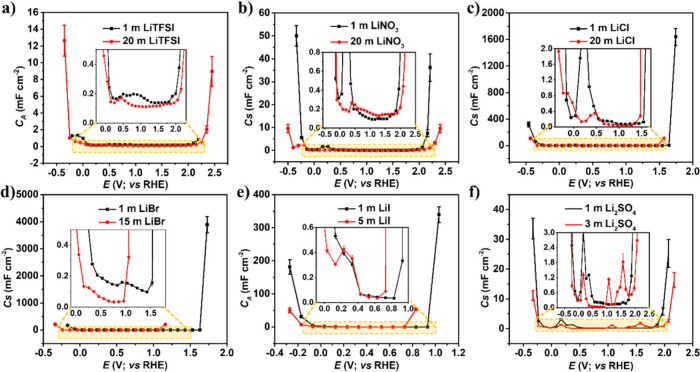
Capacitance–potential (C-E) curves of a) LiTFSI, b) LiNO_3_, c) LiCl, d) LiBr, e) LiI, and f) Li_2_SO_4_ electrolytes.

The capacitance values for all electrolytes remained relatively stable, and sudden enhancement was observed in each case as observed in Figure 4. Electrolyte-specific studies reveal a strikingly consistent pattern in supercapacitor behavior: capacitance remains stable across the midvoltage range but shows sharp enhancement at voltage extremes. For salts such as LiTFSI, LiNO_3_, and Li_2_SO_4_, capacitance scales primarily with water concentration rather than anion identity, with faradaic processes such as hydrogen and oxygen evolution or anion decomposition driving edge effects. In contrast, halides such as LiCl, LiBr, and LiI exhibit concentration-dependent capacitance, where oxidative reactions of chloride, bromide, and iodide ions produce pronounced enhancements, especially at higher molarities. Among them, iodide stands out as the most redox-active, amplifying capacitance even at modest concentrations.

This voltage-driven framework underscores that interfacial dynamics are governed less by the choice of salt and more by the applied potential and water content. For practical applications, this insight is critical: it defines safe operating voltage ranges, guides the rational selection of electrolytes, and informs strategies to balance efficiency with chemical stability. By focusing on voltage management rather than solely on anion identity, researchers and engineers can design supercapacitors and electrochemical sensors that achieve both high performance and long-term durability.

Two distinct trends were observed during the OER analysis, as seen from Figure 5. First, the overpotentials in LiTFSI, LiNO_3_, and Li_2_SO_4_ electrolyte solutions were noticeably increased compared to those in LiCl, LiBr, and LiI electrolyte solutions. In LiTFSI, LiNO_3_, and Li_2_SO_4_ electrolyte solutions, the overpotentials were independent of the electrolyte salt type but exhibited a linear relationship with the water concentration. As the water concentration decreased, meaning the electrolyte salt concentration increased, the overpotential increased. On the other hand, in LiCl, LiBr, and LiI electrolytes, the aqueous and concentrated electrolytes demonstrated a large gap in overpotentials. Importantly, in the lithium-halide-based electrolyte, the oxidation of Cl^–^, Br^–^, and I^–^ ions dominated compared to the case for the OER. Thus, with increasing salt concentration in the electrolyte, the halide oxidation took place, which is mainly responsible for achieving lower overpotentials compared to other electrolytes.

**Figure 5:**
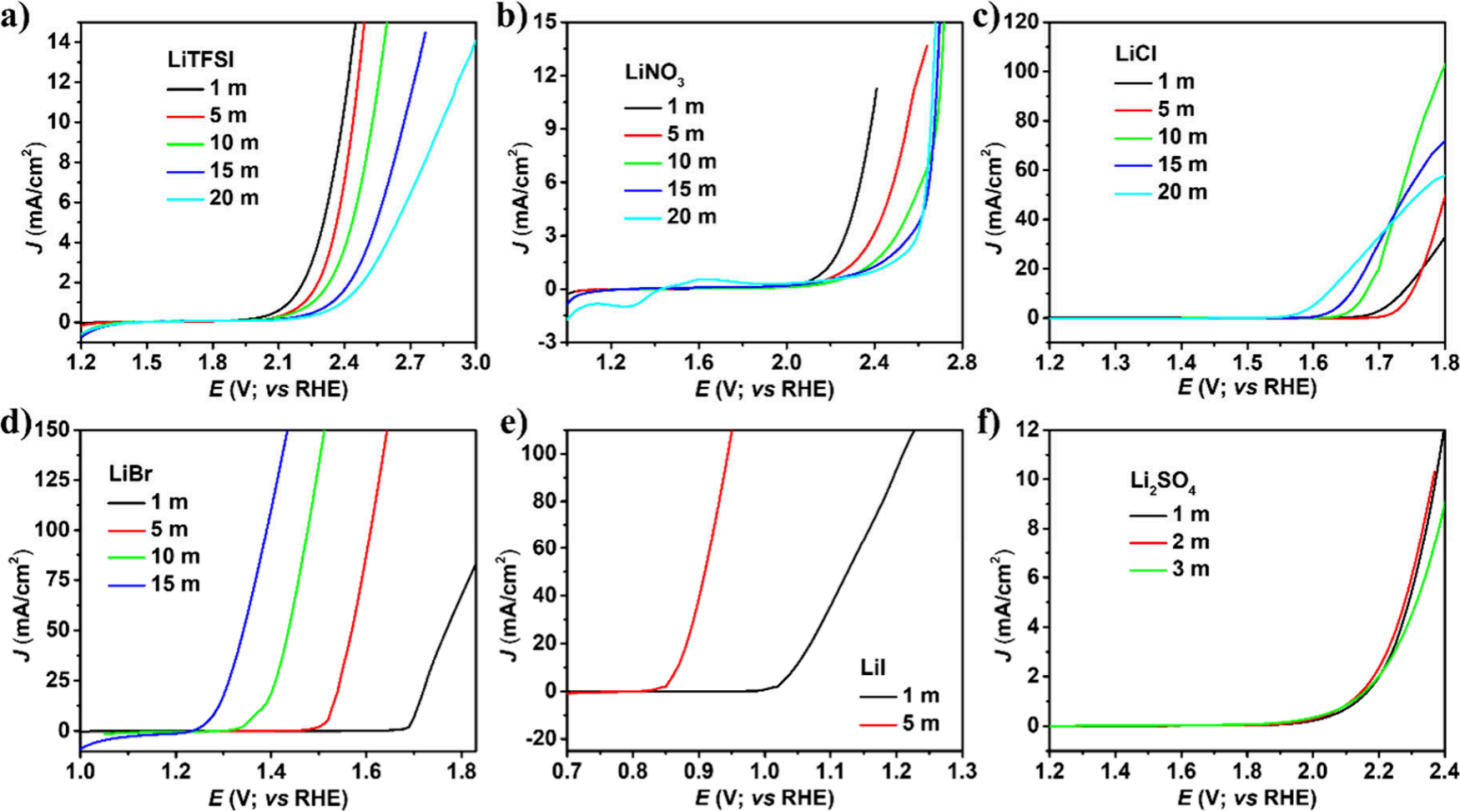
Linear sweep voltammograms of Pt electrode at scan rate 25 mV/s in different concentrations of a) LiTFSI, b) LiNO_3_, c) LiCl, d) LiBr, e) LiI, and f) Li_2_SO_4_ electrolyte.

Most of the prepared lithium-based electrolytes reveal close to neutral pH. Thus, the electrochemical OER in a neutral electrolyte involves the following key reaction in which generation of oxygen gas and release of protons into electrolytes occur as follows:

1 $$2H_{2}O\to O_{2}+4H^{+}+4e^{-}$$

Compared to aqueous electrolytes, the higher overpotential observed for LiTFSI, LiNO_3_, and Li_2_SO_4_ revealed that other side reactions including TFSI^–^, NO_3_^–^, and SO_4_^–^ oxidation reactions are not significantly favorable. At a superconcentrated level of 20 m LiNO_3_, the electrolyte reaches a voltage of 2.818 V, attributed to the decrease of water shells surrounding Li^+^ ions. In the case of LiNO_3_, after 10 m electrolytes, there was not much change observed in the LSV curve, confirming the saturation level of salt and preventing further OER performance. Studies by Zheng et al.^30^ suggest that increasing LiNO_3_ concentration significantly alters the hydration structure of Li^+^ ions—from the typical Li^+^(H_2_O)_4_ complex to polymer-like (Li^+^(H_2_O)_2_)_n_ aggregates at higher concentrations. This structural transformation enhances the electrochemical properties of superconcentrated LiNO_3_, making it a high-voltage electrolyte, comparable to LiTFSI. For Li_2_SO_4_, there was not much difference observed in the OER performance, as there is not much difference in electrolyte concentrations. The redox potential window was found to be ∼ 2.8 V vs RHE. Increasing the concentration of Li_2_SO_4_ is not beneficial, as it could deteriorate electrolyte properties, making electrode modification necessary to address performance issues. Especially, the upper limits were more expanded in LiTFSI, LiNO_3_, and Li_2_SO_4_ than the lower limits in highly concentrated electrolytes.

In LiCl, LiBr, and LiI electrolytes, the overpotential is suppressed with increasing concentration of salts due to the oxidation of chlorine, bromine, and iodine.^31^ The oxidation reaction of the LiCl electrolyte in water primarily involves the chloride ions undergoing oxidation. In an aqueous solution, LiCl dissociates into Li^+^ and Cl^–^ ions. As aqueous electrolyte, LiCl (chloride) concentration is low; OER happens as previously discussed, but at higher chloride concentration, the chloride ions are oxidized to form chlorine gas (Cl_2_), while water may also participate in oxidation reactions.^32^

2 $$2{\mathrm{Cl}}^{-}\to {\mathrm{Cl}}_{2\left(\mathrm{gas}\right)}+2e^{-}$$

It further forms hypochlorous acid (HOCl), especially under acidic to neutral pH.

3 $${\mathrm{Cl}}_{2}+H_{2}O↔\mathrm{HOCl}+H^{+}+{\mathrm{Cl}}^{-}$$

Similarly, the electrochemical oxidation reaction of the LiBr electrolyte involves the oxidation of bromide ions (Br^–^) to form bromine gas (Br_2_). Further it hydrolyzed to form hypobromous acid. At higher concentration, bromide oxidation is significant compared to OER.^33^

4 $$2{\mathrm{Br}}^{-}\to {\mathrm{Br}}_{2}+2e^{-}$$

5 $${\mathrm{Br}}_{2}+H_{2}O↔\mathrm{HOBr}+H^{+}+{\mathrm{Br}}^{-}$$

The iodine oxidation reaction in a LiI water-based electrolyte involves the conversion of iodide ions (I^–^) into iodine (I_2_) through electron loss. Afterward, it forms hypoiodous acid as shown below.^34^

6 $$2I^{-}\to I_{2}+2e^{-}$$

7 $$I_{2}+H_{2}O↔\mathrm{HOI}+H^{+}+I^{-}$$

As the concentration of LiCl, LiBr, and LiI electrolytes increases, the oxidation of chloride, bromide, and iodide ions becomes more dominant, leading to a suppression of the OER. At higher salt concentrations, halide oxidation (Cl_2_, Br_2_, and I_2_ formation) prevails over water oxidation, effectively reducing the overpotential as observed in Figure 5.

The HER performance of the in-prepared electrolytes mainly includes the reduction of water rather than lithium ions. The standard potential for Li^+^/Li^0^ is about −3.04 V vs RHE, far beyond the stability window of water. In most cases, the HER reaction is favorable as follows:

8 $$2H_{2}O_{\left(l\right)}+2e^{-}\to H_{2\left(g\right)}+2{\mathrm{OH}}_{\left(\mathrm{aq}\right)}^{-}$$

The TFSI anion is generally stable, but in highly concentrated electrolyte solutions its altered solvation environment can promote partial decomposition, contributing to solid-electrolyte interphase formation at electrode surfaces. Sulfate ions are highly stable; thus, no significant change in HER performance is observed as the sulfate concentration changes. In LiCl, LiBr, and LiI, water reduction dominates, producing hydrogen gas. The halide ions only participate in reduction if their oxidized forms (Cl_2_, Br_2_, I_2_) are present. In general, Cl^–^, Br^–^, and I^–^ ion reduction is not happening under an aqueous condition.

The complex reduction reaction involves in LiNO_3_ electrolyte, particularly, the nitrate ion (NO_3_^–^) undergoing multistep reduction reactions to form NH_3_, NO_2_^–^, N_2_, or NH_2_OH.^35^

9 $${\mathrm{NO}}_{3}^{-}+{6H}_{2}O+8e^{-}\to NH_{3}+9OH^{-}$$

10 $${\mathrm{NO}}_{3}^{-}+H_{2}O+2e^{-}\to {\mathrm{NO}}_{2}^{-}+2OH^{-}$$

11 $${\mathrm{NO}}_{3}^{-}+{3H}_{2}O+5e^{-}\to 1/2N_{2}+6OH^{-}$$

12 $${\mathrm{NO}}_{3}^{-}+{4H}_{2}O+6e^{-}\to NH_{2}\mathrm{OH}+7OH^{-}$$

At high concentrations, Li^+^, NO_3_^–^, and H_3_O^+^ ions form a unique solvation structure that facilitates the proton-coupled electron transfer from H_3_O^+^ to NO_3_^–^. This proximity boosts the efficiency of nitrate reduction while limiting water reduction.

It is revealed that the HER catalytic activity of Pt electrodes varies with respect to the salt concentration. A nonlinear trend is observed in LiTFSI, LiNO_3_, and LiCl electrolytes. In saturated electrolytes, higher overpotentials were observed for each electrolyte (Table S2). Compared to aqueous electrolytes, in 5 m of LiTFSI, LiNO_3_, and LiCl, a lower overpotential was observed for HER, and further with increasing salt concentration, the overpotential kept gradually increasing. In 1 m LiBr, the lowest onset potential was at −0.157 V vs RHE, and better overpotential close to 369 mV was observed at 10 mA/cm^2^. The change in overpotential in 1 m and highly concentrated electrolytes of LiTFSI (598 to 685 mV), LiNO_3_ (504 to 552 mV), LiCl (568 to 581 mV), LiBr (369 to 490 mV), LiI (380 to 472 mV), and Li_2_SO_4_ (517 to 562 mV) demonstrated that hydrogen production is prevented as the number of water molecules decrease in highly concentrated electrolytes.

The electrolyte concentration from dilute levels (1 m) to its solubility limit does not significantly extend the electrolyte potential window. Nevertheless, the HER overpotential shifted positively or negatively by tens to hundreds of millivolts, with a change in concentration of electrolytes. In most of the cases, HER performance decreases in superconcentrated electrolytes as observed in Figure 6. This low HER performance might be influenced by the formation of pH gradients and the absence of buffering species, which impact the local reaction conditions.

**Figure 6:**
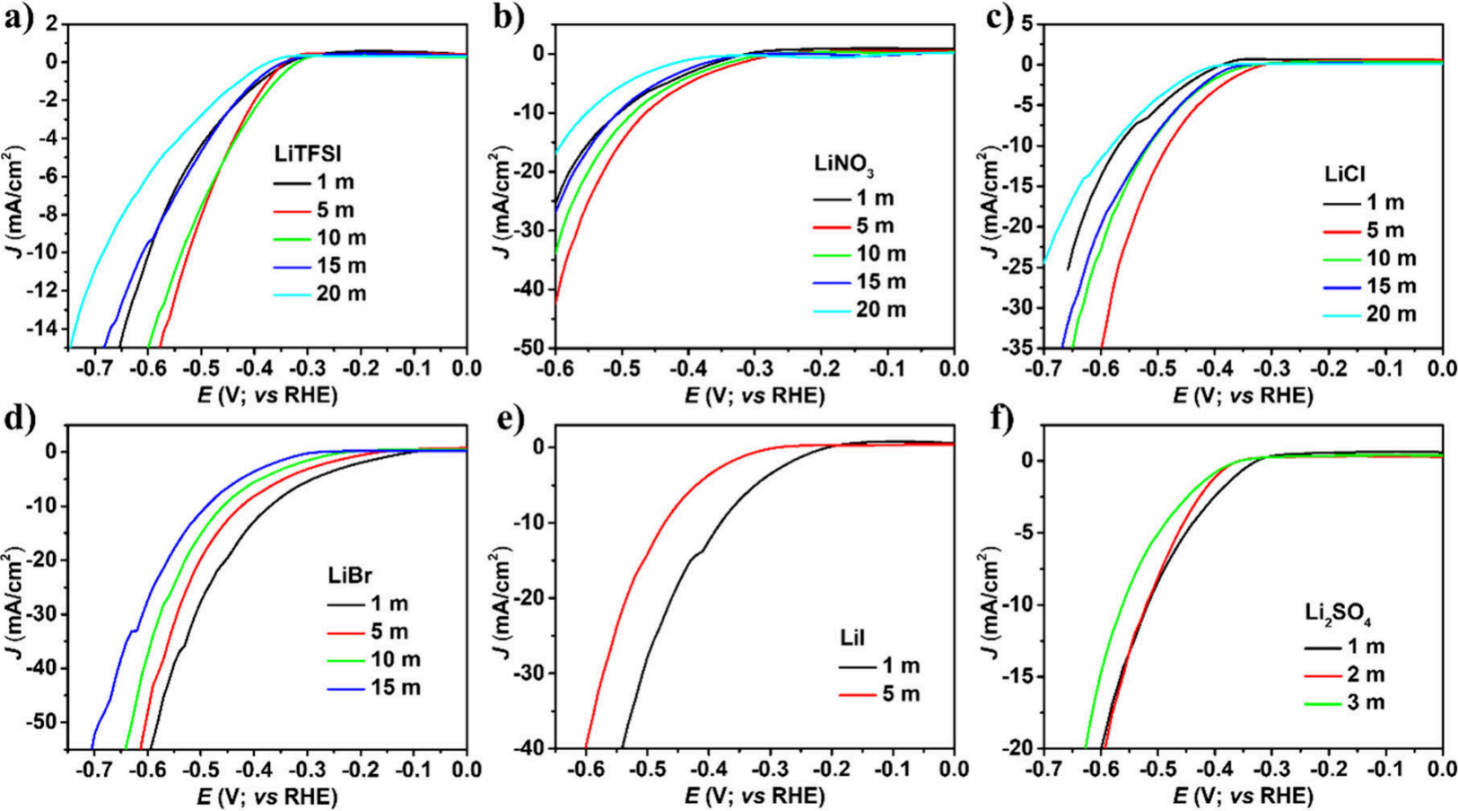
Linear sweep voltammograms of Pt electrode at scan rate 25 mV/s in different concentrations of a) LiTFSI, b) LiNO_3_, c) LiCl, d) LiBr, e) LiI, and f) Li_2_SO_4_ electrolyte.

The OER and HER onset potential regions in LSVs were used for the calculation of Tafel slopes. The change in the Tafel slope indicates a change in the OER and HER kinetics. A low Tafel slope indicates fast and efficient reaction kinetics, while a high Tafel slope indicates sluggish and inefficient reaction kinetics. The Tafel slopes for OER is lower in 1 m and higher in superconcentrated solutions of LiTFSI, LiNO_3_, and Li_2_SO_4_ (Figure S8). In the case of LiTFSI, LiNO_3_, and Li_2_SO_4_, the large, polyatomic anions (TFSI^–^, NO_3_^–^, SO_4_^2–^) are considered weakly or nonspecifically adsorbing.^36,37^ The TFSI^–^, NO_3_^–^ ions do not tend to form strong chemical bonds with the electrode surface. As confirmed through FTIR studies, in the superconcentrated electrolytes, the decrease in free water for the OER and HER is due to the formation of stable hydration shells with Li^+^, slowing down the rate-determining steps. In 1 m electrolytes, there are plenty of free water molecules. Water is a key reactant for the OER (2H_2_O → O_2_ + 4H^+^ + 4e^–^). With ample water available, the reaction can proceed through its most efficient pathway, resulting in faster kinetics and a lower Tafel slope. In superconcentrated electrolytes, most water molecules are tightly bound in the solvation shells of Li^+^ cations. The availability of free water to participate in the reaction at the electrode surface is significantly reduced. This scarcity of a primary reactant introduces a large energy barrier. The step involving water adsorption or dissociation becomes much more difficult and is likely the rate-determining step. This leads to sluggish kinetics and a higher Tafel slope.

At the positive potentials required for the OER, halide ions are strongly attracted to the electrode surface and adsorb. The halides block the active sites where water needs to bind, poisoning the surface and forcing the reaction through a less efficient pathway. This results in sluggish kinetics and a high Tafel slope. In superconcentrated electrolytes, the interfacial environment is fundamentally altered. The high halide concentration can restructure the interface and enable alternative reaction pathways that may be more efficient than just blocking site. As previously discussed, a halide ion (Cl^–^, Br^–^, I^–^) might be oxidized to an intermediate, which then chemically oxidizes water. This halide-mediated mechanism can be much faster than the direct oxidation of water on an electrode surface, leading to unexpectedly efficient kinetics and a low Tafel slope value.^38^

For HER, the trend is the same for all lithium-based electrolytes. This is because the reaction occurs at negative potentials, which minimizes the role of anion adsorption and makes water activity the dominant factor for all of the electrolytes. The Tafel slope value is lower in 1 m and higher in superconcentrated electrolytes for all electrolytes (Figure S9). At the negative potentials required for the HER, the electrode surface is negatively charged. This negative charge electrostatically repels all anions, including halides. Therefore, the strong specific adsorption of halides seen during the OER does not occur during the HER. Since strong anion adsorption is no longer a major factor, the kinetics for all salts are now governed by the same thing: water activity. Water is abundant. HER, which uses water as a reactant (2H_2_O + 2e^–^ → H_2_ + 2OH^–^), proceeds efficiently in 1 m of electrolytes. This results in lower Tafel slopes. Whereas in superconcentrated electrolytes, water is scarce. The lack of the water reactant hinders the reaction kinetics for all the salts equally, resulting in a high Tafel slope value.

As LiTFSI and LiNO_3_ concentration increases from 1 m to 5 m, the pH rises while the OER overpotential increases. This trend shows that higher alkalinity does not improve OER efficiency. Beyond 10 m, the pH drops steadily, yet the overpotential slightly decreases, suggesting other factors affect performance. Although conductivity peaks at 5 m in the case of LiTFSI and 10 m LiNO_3_, the OER overpotential also peaks there, implying limitations beyond ionic mobility. These effects may include ion crowding, reduced water activity, and changes in the electrode interface that hinder catalytic efficiency. In the case of HER, at moderate salt concentrations, slightly alkaline pH and high conductivity support the most efficient HER performance on Pt electrodes. As salt concentration increases further, interfacial factors like ion crowding and reduced proton availability overpower conductivity benefits, leading to higher overpotentials.

The mechanistic origin of the observed redox behavior in water-in-salt electrolytes can be rationalized by considering three interrelated factors: specific anions preferentially adsorb at the electrode surface, forming an anion-rich interfacial layer that displaces water and delays oxygen evolution, providing kinetic protection and altering interfacial capacitance. This behavior is supported by anion surface excess and potential-driven switching between anion- and water-rich interfaces.^39^ Concurrently, the electric double layer is restructured with fewer fully hydrated Li^+^ ions and increased ion clustering, leading to modified desolvation energetics, solid-electrolyte interface precursor formation, and asymmetric capacitance, as evidenced by voltage-dependent clustering and nonideal electrical double-layer behavior predicted by theory and molecular dynamics.^40^ At the same time, the overall reduction of free water in both bulk and interfacial regions limits water availability as a reactant, suppressing water splitting and expanding the electrochemical stability window through combined thermodynamic and kinetic effects, consistent with experimental and simulation observations.^41^

With increasing concentrations of LiCl, LiBr, and LiI, the pH drops from strongly alkaline toward mildly acidic levels, accompanied by a notable reduction in the overpotential for halide oxidation. This suggests that lower pH conditions in this system enhance the reaction kinetics and improve the catalytic efficiency. Conductivity rises sharply at moderate concentrations and then declines, yet the overpotential continues to fall. This behavior indicates that interfacial effects dominate bulk ionic transport at higher concentrations. In the case of HER performance of LiCl, LiBr, and LiI, HER efficiency on Pt is better under alkaline conditions. Although conductivity peaks at 5 m and then declines, the overpotential continues to increase, pointing to limitations beyond ionic transport. Factors like chloride, bromide, and iodide ion adsorption and altered water structure may contribute to the decrease in overpotential performance observed in concentrated LiCl, LiBr, and LiI.

## Conclusions

The potential window and the OER and HER analyses of various lithium-based electrolytes were investigated using a platinum electrode. The hydrogen and hydroxyl bonding in the aqueous to superconcentrated lithium-based electrolytes helps us to understand their unique solvation as characterized by FTIR. CV and LSV were used to understand redox reactions, kinetics, and reaction overpotentials. The interaction between platinum and highly concentrated aqueous electrolyte environments is explored to understand its impact on the catalytic activity of the OER/HER. The difference for the overpotentials of the OER was much higher than that for the HER overpotentials. The HER overpotentials were not affected with respect to the concentration of electrolytes. Lithium nitrate can be a challenging candidate for replacement of LiTFSI, as it shows almost a similar potential window. Lithium halide-based salts accelerate redox processes at “water-in-salt” concentrations but do not expand the voltage window, making them ideal as redox additives. This study broadens the potential for investigating various lithium-based electrolytes for energy conversion and storage applications using superconcentrated electrolytes.

## Supplementary Material



## Notes

Experimental details on materials, electrolyte preparation, and electrochemical measurements. Figures S1–S9 present physical properties, FTIR data, contact angle, EIS spectra, Bode plot and Tafel slope of various lithium-based electrolytes. Table S2 compares onset potentials and overpotentials from LSV analyses (PDF)
