# Supplementary material for: Anion-Dependent Redox Pathways Governing Water Splitting in Superconcentrated Lithium Electrolytes
Source: ACS Phys Chem Au. 2026 Apr 22;6(3):425–34. doi: 10.1021/acsphyschemau.6c00037 (PMC13220191; doi:10.1021/acsphyschemau.6c00037)
Supplement: Supplementary file 1 [file pg6c00037_si_001.pdf]

Supporting information

Anion-Dependent Redox Pathways Governing Water Splitting in Superconcentrated Lithium Electrolytes

Sagar Ingavale[a, b], *Pawin Iamprasertkun*[a, b, *]

[a]School of Bio-Chemical Engineering and Technology, Sirindhorn International Institute of Technology, Thammasat University, Pathum Thani 12120, Thailand

[b]Research Unit in Sustainable Electrochemical Intelligent, Thammasat University, Pathum Thani 12120, Thailand

*Corresponding author Email: pawin@siit.tu.ac.th

CONTENTS

1. Experimental details
    1.1 Materials
    1.2 Table S1-Details of electrolyte preparation
    1.3 Electrochemical measurements
2. Figures

    Figure S1: The physical properties of the various electrolytes at different concentrations: (a) pH, and (b) electrical conductivity.

    Figure S2: ATR-FTIR spectra of H-OH bending and H-OH stretching, of different concentrations of a) LiTFSI, b) $LiNO_3$, c) LiCl, d) LiBr, e) LiI, and f) Li_2SO_4 electrolyte.

    Figure S3: The contact angle measurement of various lithium-based electrolytes with respect to the electrolyte concentration.

    Figure S4: The Nyquist plot of the Pt electrode in different concentration of (a-b) 1 and 20 m LiTFSI, (c-d) 1 and 20 m $LiNO_3$, and, (e-f) 1 and 20 m LiCl, electrolytes.

    Figure S5: The Nyquist plot of the Pt electrode in different concentration of (a-b) 1 and 15 m LiBr, (c-d) 1 and 5 m LiI, and, (e-f) 1 and 3 m Li_2SO_4 electrolytes

    Figure S6: The Bode plots of the Pt electrode in different concentration of (a-b) 1 and 20 m LiTFSI, (c-d) 1 and 20 m $LiNO_3$, and, (e-f) 1 and 20 m LiCl, electrolytes.

    Figure S7: The Bode plots of the Pt electrode in different concentration of (a-b) 1 and 15 m LiBr, (c-d) 1 and 5 m LiI, and, (e-f) 1 and 3 m Li_2SO_4 electrolytes.

    Figure S8: Comparative OER Tafel slope at different concentrations of a) LiTFSI, b) $LiNO_3$, c) LiCl, d) LiBr, e) LiI, and f) Li_2SO_4 electrolytes.

    Figure S9: Comparative HER Tafel slope at different concentrations of a) LiTFSI, b) $LiNO_3$, c) LiCl, d) LiBr, e) LiI, and f) Li_2SO_4 electrolytes.

3. **Table S2:** Onset and overpotential comparison from LSVs of lithium-based electrolytes.
    Table S3: The potential and capacitance of the platinum electrode in LiTFSI and $LiNO_3$ electrolytes.

1. Experimental details

1.1 Materials

Lithium bromide (LiBr, 99% purity), lithium iodide (LiI, 99% purity), lithium nitrate ($LiNO_3$, 99.9% purity), and lithium sulfate (Li_2SO_4, 99.9% purity) were obtained from Acros, while lithium chloride (LiCl, 98% purity) was obtained from Kemaus. Lithium bis(trifluoromethanesulfonyl) imide (LiTFSI, 99.9% purity) was procured from Sigma-Aldrich. Electrolyte solutions were prepared based on molality (mol kg^{-1}), abbreviated as [m]. The aqueous electrolytes were prepared using deionized water. The electrolyte concentrations were set as follows: LiBr: (1 m, 5 m, 10 m, and 15 m), LiCl, $LiNO_3$, and LiTFSI: 1 m, 5 m, 10 m, 15 m, and 20 m, Li_2SO_4: 1 m, 2 m, and 3 m, LiI: 1 m and 5 m.

1.2 **Table S1:** Details of electrolyte preparation

Salt	Molality (m)	Salt (g)	Water (ml)
LiTFSI	1	1.43545	5
	5	7.1772	5
	10	14.3545	5
	15	21.532	5
	20	28.709	5
$LiNO_3$	1	0.3447	5
	5	1.7235	5
	10	3.447	5
	15	5.1705	5
	20	6.894	5
LiCl	1	0.21195	5
	5	1.05975	5
	10	2.1195	5
	15	3.17925	5
	20	4.239	5
LiBr	1	0.4342	5
	5	2.1711	5
	10	4.3422	5
	15	6.5133	5
LiI	1	0.66925	5
	5	3.346	5
Li_2SO_4	1	0.5497	5
	2	1.0994	5
	3	1.6491	5

1.3 Electrochemical measurements

For electrochemical investigation, a three-electrode setup was employed at room temperature. The platinum disc electrode is cleaned by polishing with 1 μm alumina, thorough rinsing with deionized water. The cleaning of platinum wire involves sequential rinsing with water and ethanol, followed by sonication and annealing. After cooling down, it was washed again with water. The measurements were carried out using a platinum disc electrode with a geometric surface area of 0.0314 cm^2, serving as the working electrodes. The current densities reported in the present studies were normalized with the surface area of Pt. (To calculate the geometric surface area of a platinum disc with a 2 mm diameter: Area (A) = $\pi \times r^2 = \pi \times (0.1\ cm)^2 = \pi \times 0.01\ cm^2 = 0.0314\ cm^2$). In this setup, a platinum wire was utilized as the counter electrode, while a silver/silver chloride (Ag/AgCl) electrode served as the reference electrode. Finally, the electrode potentials were converted to the reversible hydrogen electrode (RHE) using $E_{RHE} = E_{Ag/AgCl} + 0.059(pH) + E^{o}_{RHE}$. The CV and LSV techniques were tested in the prepared electrolytes saturated with nitrogen (N_2). The saturation process was ensured by purging the N_2 gas for 15 minutes.

At the beginning, cyclic voltammogram stabilized using several cycles. The CV was recorded by scanning the potential in positive and negative directions to achieve an arbitrarily current density of about 1-10 mA/cm^2. The cyclic voltammograms of Pt electrode studied at scan rate 100 mV s^{-1} in different concentrations of a) LiTFSI, b) $LiNO_3$, c) LiCl, d) LiBr, e) LiI, and f) Li_2SO_4 electrolytes. The potential region selected for Tafel slope extraction corresponds to the Faradaic portion of the LSV, beyond the onset of the oxygen evolution reaction (OER), where current increases exponentially due to charge-transfer kinetics. Linear sweep voltammograms of Pt electrode at scan rate 25 mV/s in different concentrations of a) LiTFSI, b) $LiNO_3$, c) LiCl, d) LiBr, e) LiI, and f) Li_2SO_4 electrolyte.

Capacitance calculation

To examine the capacitance-potential characteristics of platinum electrode in various electrolytes of varying concentrations, the interfacial capacitance (C_A) was evaluated by averaging the capacitance values obtained within the frequency range of 0.1 to 100 KHz. This range corresponds to conditions under which the phase angle approaches −90°, indicative of predominant capacitive behavior, using following equation.

$$C_A = -1 / 2\pi f Z'' A \qquad \text{S1}$$

where f is the applied frequency (Hz), Z'' is the imaginary component of the impedance, and

A is the surface area of the platinum.

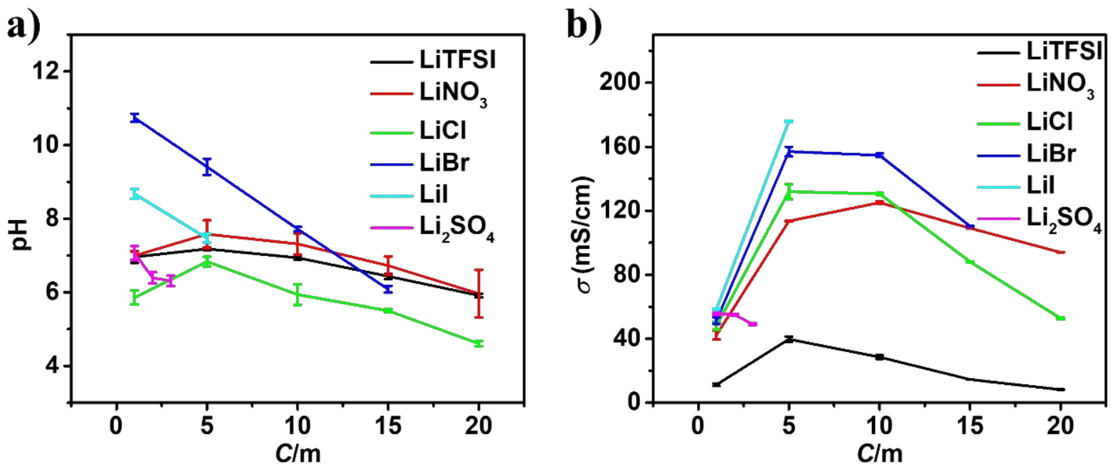

Figure S1: The physical properties of the various electrolytes at different concentrations: (a) pH, and (b) electrical conductivity.

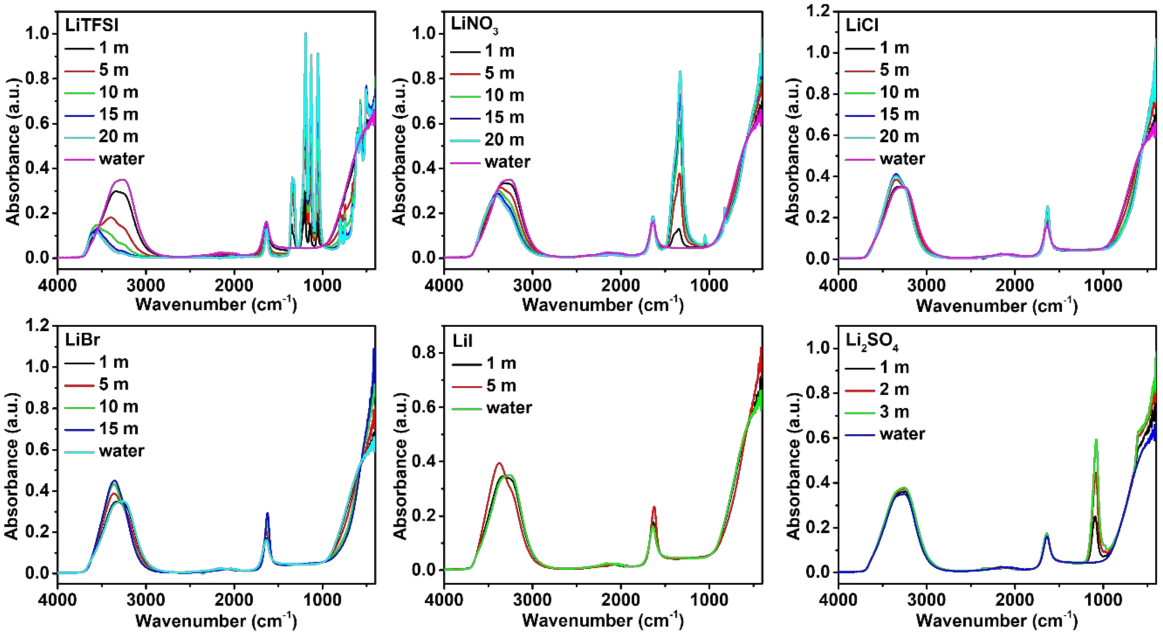

Figure S2: ATR-FTIR spectra of H-OH bending and H-OH stretching, of different concentrations of a) LiTFSI, b) $LiNO_3$, c) LiCl, d) LiBr, e) LiI, and f) Li_2SO_4 electrolyte.

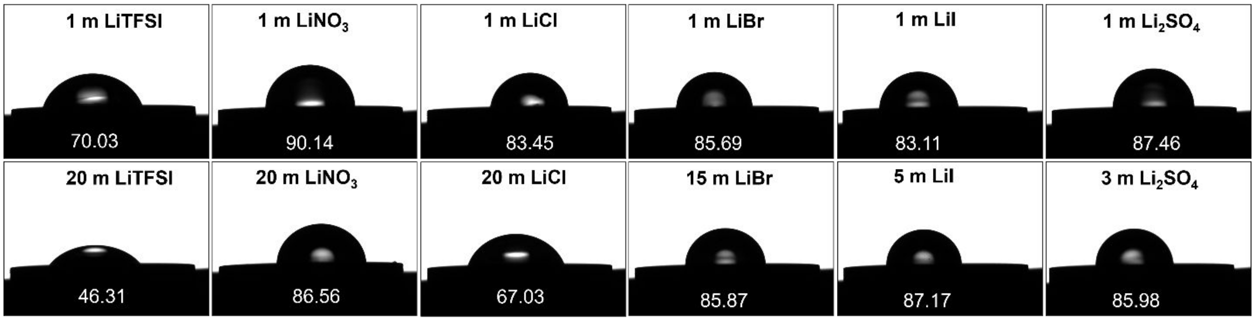

Figure S3: The contact angle measurement of various lithium-based electrolytes with respect to the electrolyte concentration.

Electrochemical impedance spectroscopy (EIS) was conducted on platinum electrode in various lithium-based electrolytes, ranging from 100 kHz to 0.1 Hz. Both 1 m and the maximum concentration of each electrolyte were considered in the EIS analysis. The goal was to determine the stable potential window of each electrolyte, as illustrated in Figure 4. A key indicator of stability is when the phase angle between current and voltage approaches −90°, which reflects ideal capacitive behavior. When the phase angle begins to diverge from this value at a given potential, it signals the boundary of the electrolytes stable operating range.

For the LiTFSI electrolyte, the stable potential window at 1 m is approximately in between -0.2 V to 2.2 V versus RHE. As the concentration of LiTFSI increases to 20 m, the potential window broadens. At 20 m LiTFSI, the window expands from −0.25 V to 2.35 V versus RHE, exceeding the thermodynamic stability limits of water. Similarly, the potential window broadens in $LiNO_3$ and Li_2SO_4 electrolytes at their maximum concentrations compared to their respective 1 m solutions. In contrast, a shrinking of the potential window is observed in LiCl, LiBr, and LiI electrolytes. This behavior aligns with the redox characteristics of halide ions and is consistent with the results obtained from CV and LSV analysis.

Additionally, Nyquist plots for platinum at various lithium-based electrolytes are presented in Figure S3 & 4. These plots closely align with the Bode plots shown in Figure S5 & 6, confirming consistency between the two representations. At the stable potential across all electrolyte concentrations, the Nyquist plots do not exhibit a perfectly vertical line, suggesting deviations from ideal RC circuit behavior typically associated with purely capacitive systems. Instead, the presence of a tilted or curved line indicates contributions from resistive elements or non-ideal double-layer formation. As the potential moves beyond the double-layer region, these deviations become more pronounced, reflecting the onset of faradaic reactions and increased charge transfer activity at the electrode-electrolyte interface. Consequently, the capacitance values obtained from EIS measurements are calculated using Equation S1 and are illustrated in Figure 4 and 5.

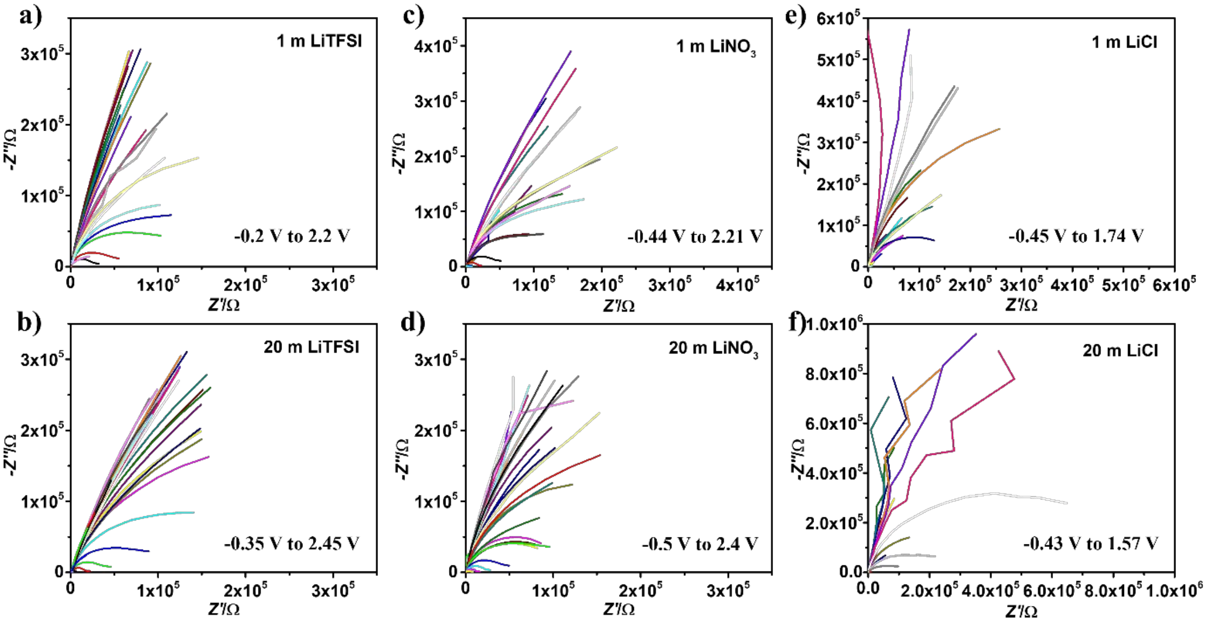

Figure S4: The Nyquist plot of the Pt electrode in different concentration of (a-b) 1 and 20 m LiTFSI, (c-d) 1 and 20 m $LiNO_3$, and, (e-f) 1 and 20 m LiCl, electrolytes.

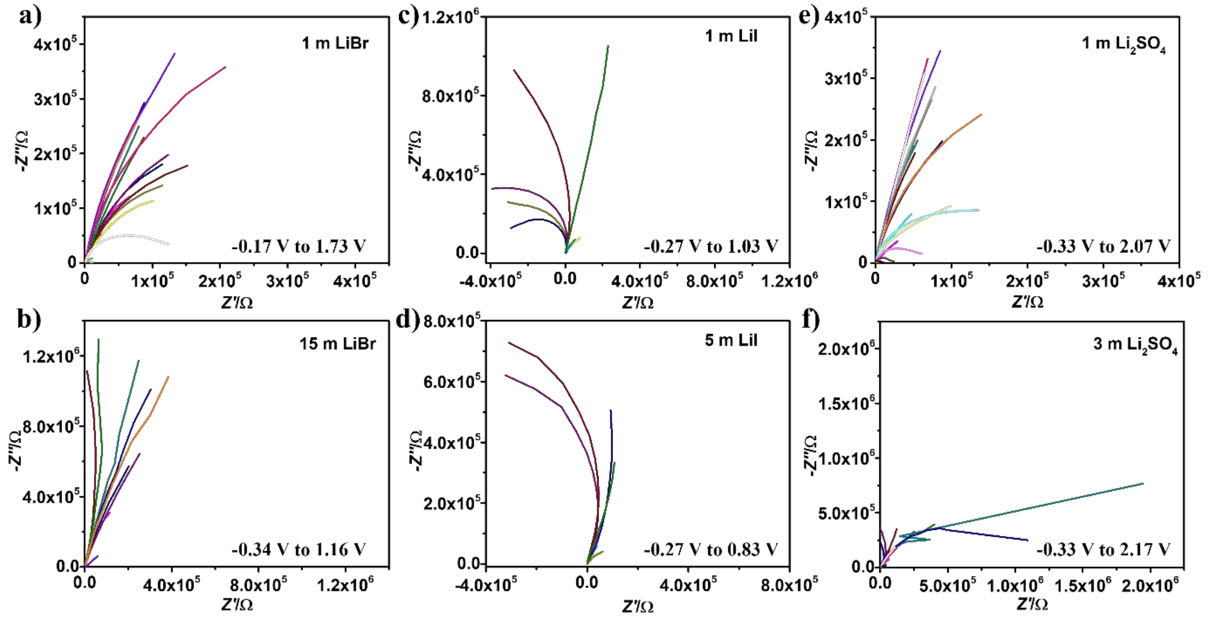

Figure S5: The Nyquist plot of the Pt electrode in different concentration of (a-b) 1 and 15 m LiBr, (c-d) 1 and 5 m LiI, and, (e-f) 1 and 3 m Li_2SO_4 electrolytes.

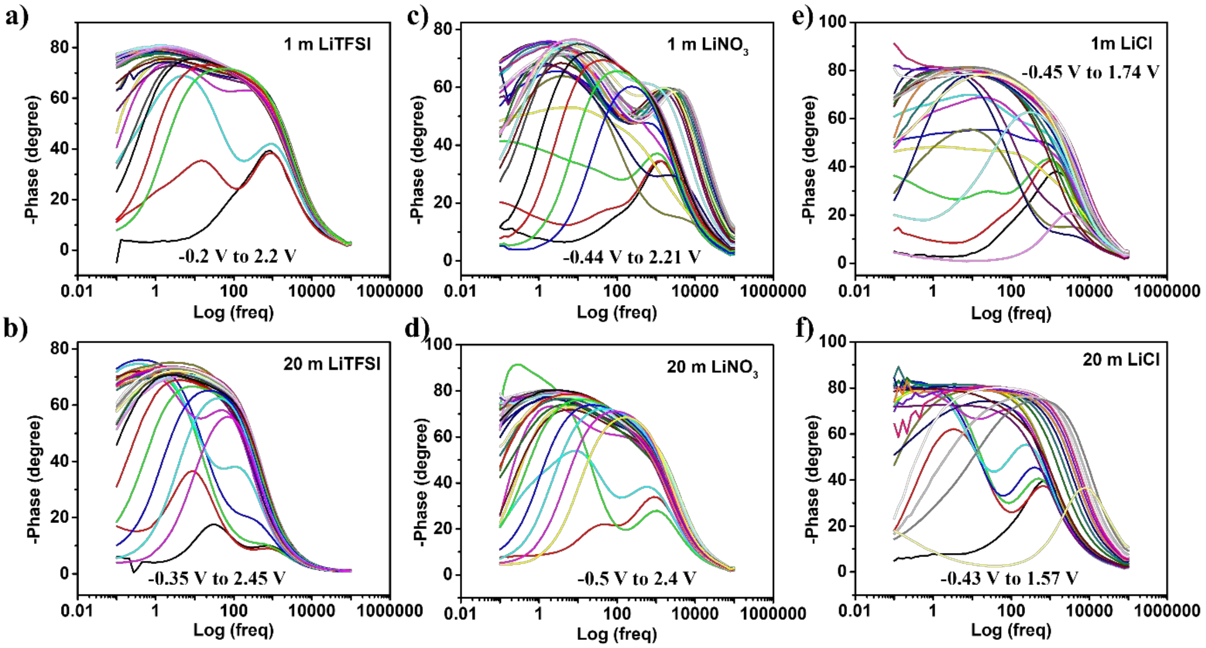

Figure S6: The Bode plots of the Pt electrode in different concentration of (a-b) 1 and 20 m LiTFSI, (c-d) 1 and 20 m $LiNO_3$, and, (e-f) 1 and 20 m LiCl, electrolytes.

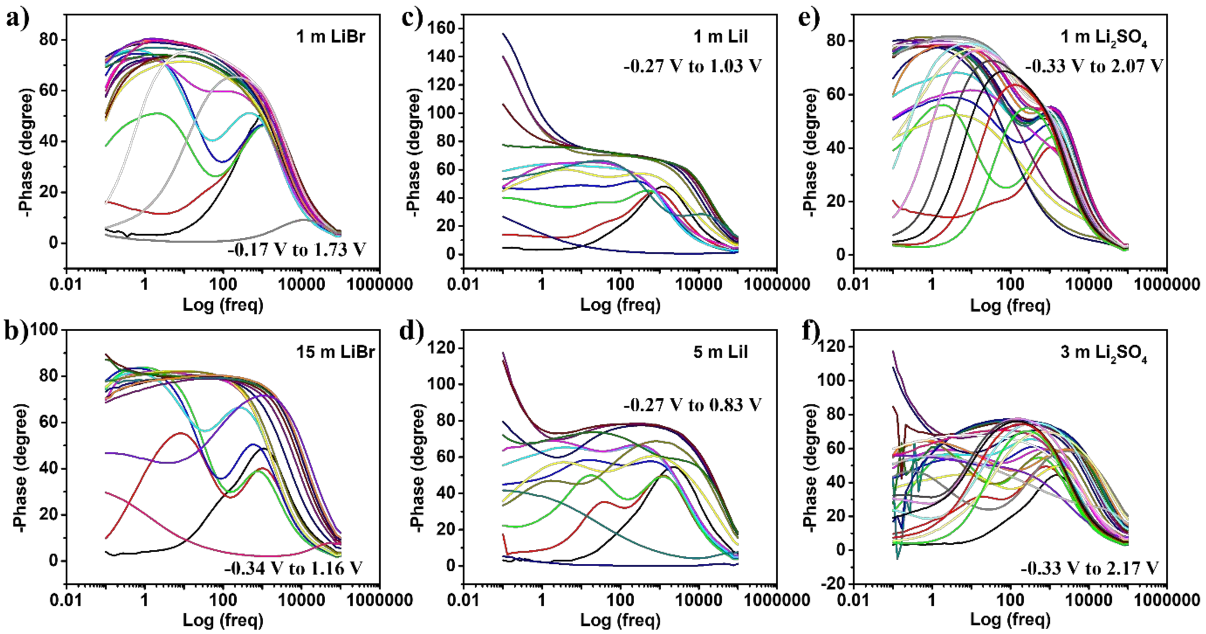

Figure S7: The Bode plots of the Pt electrode in different concentration of (a-b) 1 and 15 m LiBr, (c-d) 1 and 5 m LiI, and, (e-f) 1 and 3 m Li_2SO_4 electrolytes.

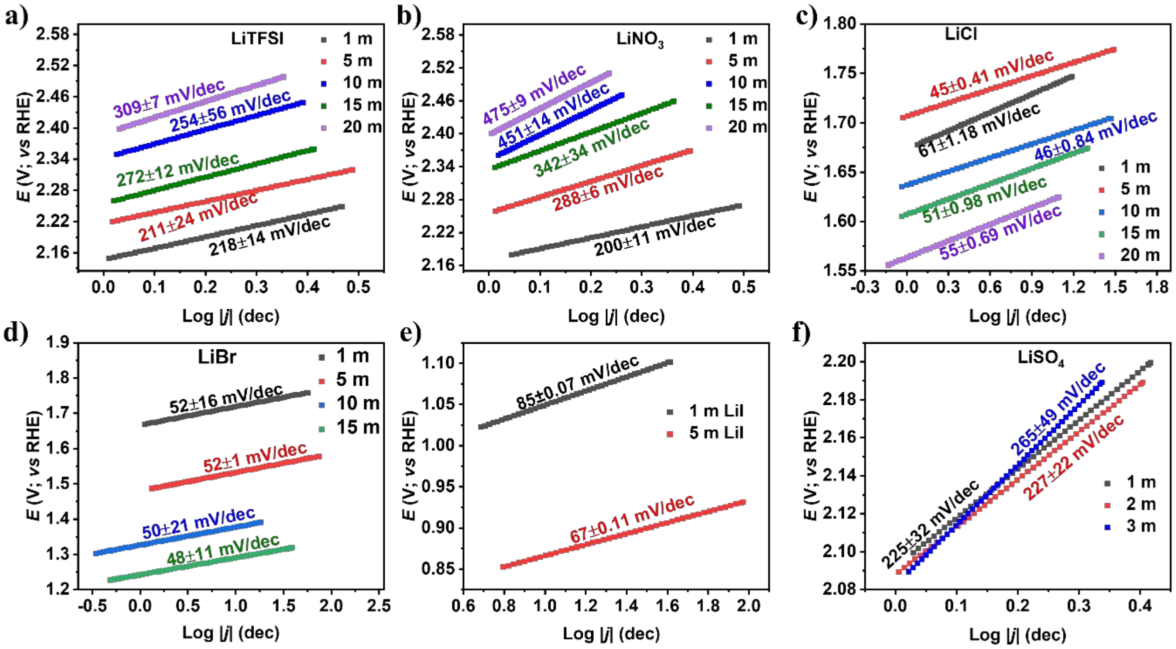

Figure S8: Comparative OER Tafel slope at different concentrations of a) LiTFSI, b) $LiNO_3$, c) LiCl, d) LiBr, e) LiI, and f) Li_2SO_4 electrolytes.

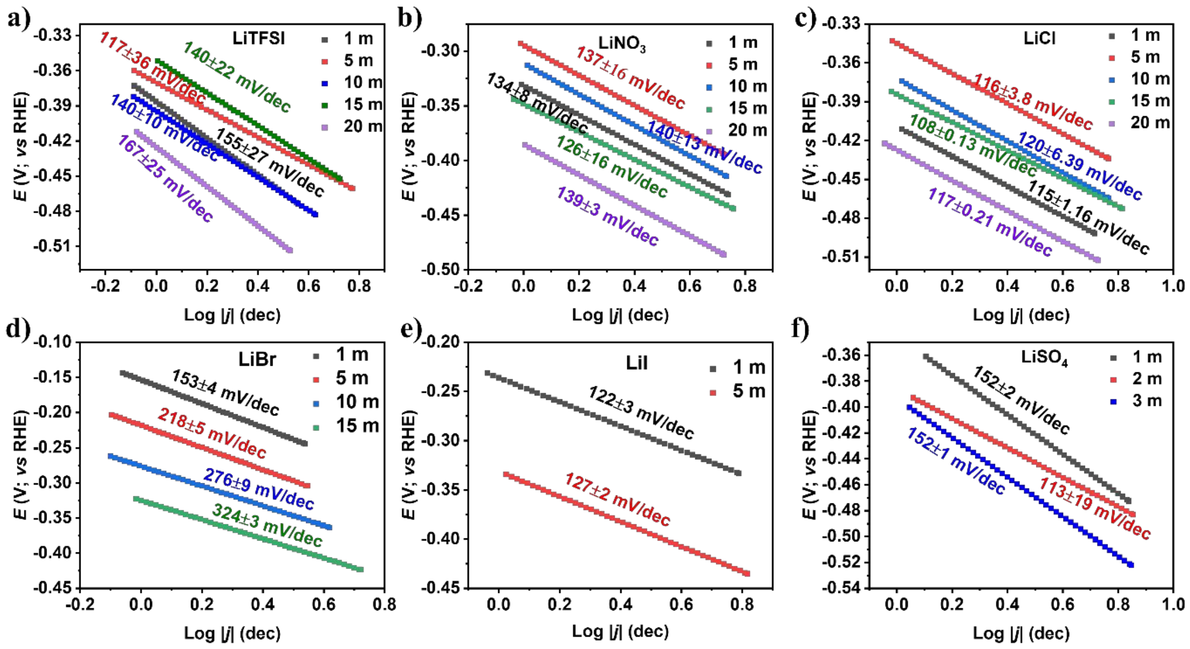

Figure S9: Comparative HER Tafel slope at different concentrations of a) LiTFSI, b) $LiNO_3$, c) LiCl, d) LiBr, e) LiI, and f) Li_2SO_4 electrolytes.

Table S2: Onset and overpotential comparison from LSVs of lithium-based electrolytes

Sr. No.	Electrolyte	OER Onset potential (V; *vs* RHE)	OER Overpotential (V; *vs* RHE)	HER Onset potential (V; *vs* RHE)	HER Overpotential (V; *vs* RHE)
1	1 m LiTFSI	2.14±0.13	1.15±0.02	-0.389±0.001	0.598±0.04
2	5 m LiTFSI	2.22±0.03	1.21±0.02	-0.376±0.004	0.524±0.02
3	10 m LiTFSI	2.26±0.06	1.29±0.01	-0.361±0.001	0.537±0.01
4	15 m LiTFSI	2.34±0.01	1.43±0.01	-0.398±0.002	0.611±0.01
5	20 m LiTFSI	2.39±0.01	1.61±0.01	-0.428±0.008	0.685±0.01
6	1 m $LiNO_3$	2.17±0.02	1.16±0.01	-0.345±0.005	0.504±0.01
7	5 m $LiNO_3$	2.25±0.01	1.33±0.01	-0.303±0.003	0.464±0.01
8	10 m $LiNO_3$	2.33±0.01	1.46±0.01	-0.323±0.003	0.483±0.01
9	15 m $LiNO_3$	2.35±0.09	1.44±0.01	-0.356±0.001	0.505±0.01
10	20 m $LiNO_3$	2.39±0.06	1.43±0.01	-0.40±0.01	0.552±0.01
11	1 m LiCl	1.67±0.01	0.50±0.01	-0.416±0.004	0.568±0.01

12	5 m LiCl	1.71±0.001	0.51±0.01	-0.351±0.001	0.483±0.01
13	10 m LiCl	1.64±0.001	0.44±0.01	-0.379±0.001	0.516±0.02
14	15 m LiCl	1.61±0.001	0.42±0.01	-0.391±0.001	0.518±0.01
15	20 m LiCl	1.56±0.001	0.39±0.01	-0.434±0.004	0.581±0.01
16	1 m LiBr	1.67±0.005	0.47±0.01	-0.157±0.013	0.369±0.02
17	5 m LiBr	1.48±0.011	0.29±0.01	-0.220±0.001	0.424±0.01
18	10 m LiBr	1.32±0.001	0.14±0.01	-0.280±0.005	0.458±0.01
19	15 m LiBr	1.24±0.005	0.05±0.01	-0.333±0.023	0.490±0.01
20	1 m LiI	1.00±0.001	-0.18±0.01	-0.242±0.012	0.380±0.02
21	3 m LiI	0.84±0.006	-0.36±0.01	-0.339±0.029	0.472±0.02
22	1 m Li_2SO_4	2.13±0.03	1.14±0.01	-0.363±0.013	0.517±0.01
23	2 m Li_2SO_4	2.11±0.02	1.13±0.01	-0.396±0.004	0.519±0.01
24	3 m Li_2SO_4	2.11±0.02	1.18±0.01	-0.408±0.003	0.562±0.01
